# Editorial: Perspectives of Antiarrhythmic Drug Therapy: Disappointing Past, Current Efforts, and Faint Hopes

**DOI:** 10.3389/fphar.2020.01116

**Published:** 2020-07-22

**Authors:** Péter P. Nánási, Esther Pueyo, László Virág

**Affiliations:** ^1^Department of Physiology, University of Debrecen, Debrecen, Hungary; ^2^BSICoS Group, Aragón Institute of Engineering Research, University of Zaragoza, IIS Aragón and CIBER-BBN, Zaragoza, Spain; ^3^Department of Pharmacology and Pharmacotherapy, University of Szeged, Szeged, Hungary

**Keywords:** antiarrhythmic agents, antiarrhythmic strategies, proarrhythmic mechanisms, cardiac ion currents, cardiac arrhythmias

Present issue is devoted to those areas of cardiac electrophysiology, pathophysiology, and pharmacology which are critically important to improve the efficacy of antiarrhythmic treatment. Cardiac arrhythmia is a leading cause of morbidity and mortality, and the history of anti-arrhythmic drug therapies has been dismal. Development of apparently more and more selective and effective antiarrhythmic agents has been in the focus of interest of drug research in the past few decades, however, neither the ideal compound, nor an optimal strategy could be demonstrated so far. Furthermore, disappointing results have been obtained with several old antiarrhythmic agents, like many class I/C and class III drugs as documented by the CAST (Investigators of the Cardiac Arrhythmia Suppression Trial Preliminary report, 1989) and SWORD (Waldo et al., 1995) studies, respectively. The direct proarrhythmic side-effects of many currently applied antiarrhythmic agents are evident by now. Some “malpractices” in the past, however, could have been prevented by studying the actions of conventional antiarrhythmic compounds systematically in a frequency-dependent manner. One approach may be studying the kinetics of electrical restitution of action potential duration (APD) as presented by Árpadffy-Lovas et al. in this issue. They studied the restitution kinetics of APD in many species, including humans, and concluded that in addition to basic APD, some ion currents, like I_Na_ or I_to_, may also modify restitution kinetics. Due to the insufficient therapeutic results in the past we are continuously trying to find new trails, new antiarrhythmic mechanisms (each of them, of course, will also carry its own pitfalls), such as the suppression of late Na^+^ current, application of Na^+^/Ca^2+^ exchanger blockers, or selective manipulation of CaMKII activity. All these ideas are discussed in the present issue in details.

Better understanding of the proarrhythmic mechanisms may minimize the unwanted side-effects and yield better therapeutic results. Accordingly, having a deeper insight into the properties of the finely tuned myocardial Ca^2+^-handling, its regulatory and modulatory role in the entire cardiac electrogenesis, including action potential morphology, pacemaker activity, and arrhythmogenesis, may allow the development of novel antiarrhythmic strategies. The role of pathological Ca^2+^ handling in development of cardiac arrhythmias is reviewed in this issue by Kistamás et al. The most important target of intracellular Ca^2+^ is the calcium-calmodulin kinase II (CaMKII), which is known to influence several Ca^2+^ sensitive ion channels. Therefore, it is not surprising that modification of CaMKII activity is a new and promising therapeutic strategy as presented by Nassal et al. in this issue. In addition to activation of CaMKII, changes of intracellular Ca^2+^ concentration and transmembrane Ca^2+^ fluxes have emerging influence on pacemaker activity through the operation of the Na^+^/Ca^2+^ exchanger, as we learn from two articles in this issue—both from Kohajda et al. These studies focus on interaction between the “membrane” clock” and “calcium clock”, mechanisms which are believed to stabilize pacemaker activity of nodal cells (Tsutsui et al., 2018).

Since cardiac electrogenesis is based on the well balanced activity of ion channels mediating inward and outward currents, development of sustained inward currents during the plateau, seen frequently in the remodeled myopathic hearts, is clearly proarrhythmic due to the increased incidence of early afterdepolarizations. Therefore, studies on the late Na^+^ current, including its CaMKII-dependent regulation, as presented by Horváth et al. in this issue, is a new promising area of antiarrhythmic research. Here is to be mentioned that the antiarrhythmic efficacy of ranolazine, the well-known inhibitor of the late Na^+^ current, is partially attributable to its inhibitory potency on TASK1 K^+^ channels, as reported by Ratte et al. in this issue, concluding that “this puts forward ranolazine as a prototype drug for the treatment of atrial arrhythmia because of its combined efficacy on atrial electrophysiology and lower risk for ventricular side effects”. Further approach in the treatment of persistent atrial fibrillation may be the inhibition of other K^+^ currents, like the background inward rectifier (I_K1_) or the acetylcholine-activated inward rectifier (I_K,ACh_). Both currents are blocked by the well-known antimalarial agent, chloroquine (which may probably be useful against the new COVID pandemy as well). Importantly, the efficacy against atrial fibrillation correlated with K^+^ current inhibiting potencies of the drug, as reported by Tobón et al. in this issue. In addition to electrophysiological interventions, supporting the mechanical performance of the heart is also part of modern treatment of atrial fibrillation. According to the report of Suo et al. in this issue, the angiotensin receptor inhibitor valsartan displayed beneficial effects in cases of atrial fibrillation and decreased the level of heart failure.

Cardiac arrhythmias are often consequences of mutations targeting ion channels of the surface membrane. Thus, expression and investigation of abnormal (mutant) ion channels, as well as determination of the critical molecular segments may help to develop more effective antiarrhythmic practices, while this track represents new challenges as well. Since the abnormal ion channels may be also potential drug targets, their investigation in transgenic animal models is a promising new field of research as discussed by Baczko et al. in this issue. In addition to *in vivo* modeling, *in silico* modeling of ion channel gating, arrhythmia incidence, and antiarrhythmic drug action may further improve our positions in the antiarrhythmic offensive. Examples for this are provided by Zhou et al. and Horgmo Jaeger et al.-both studies are presented in this issue.

Two further review articles in this issue have to be mentioned. The one by Tosaki gives a general picture vertically from the molecular mechanisms of cardiac arrhythmias to the new antiarrhythmic strategies. The other by Szabó et al. does the same from the aspect of the clinical practice in terms of emergency medicine focusing on the problem of sudden cardiac death. Both papers give full overview of the current stage of antiarrhythmic treatment.

Finally, when developing new antiarrhythmic strategies, based on the suppression or activation of a cardiac ion current, it has to be born in mind that the evolution had time enough to elaborate the optimal duration and morphology of the cardiac action potential (optimal, of course, under physiological conditions only) and their modification may worsen the arrhythmia incidence. As a consequence, we can find only better, but not ideal, new drugs and strategies. Probably the most attractive solution in the future should be the general introduction of personalized antiarrhythmic treatment, which may improve our chances against life threatening cardiac arrhythmias.

## Author Contributions

The authors confirm being the contributors of this work and approved it for publication.

## Funding

This work was supported by the European Research Council under grant agreement ERC-StG 638284, by Ministerio de Ciencia e Innovación (Spain) through project PID2019-105674RB-I00 and by European Social Fund (EU) and Aragón Government through BSICoS group (T39\_20R) and project LMP124-18.

## Conflict of Interest

The authors declare that the research was conducted in the absence of any commercial or financial relationships that could be construed as a potential conflict of interest.
